# Early Hearing Interventions for Children with Hearing Loss in Africa: A 21-Year Scoping Review (2004–2025)

**DOI:** 10.3390/children12070864

**Published:** 2025-06-30

**Authors:** Stavros Hatzopoulos, Ludovica Cardinali, Piotr Henryk Skarzynski, Abiodun T. Adewunmi, Giovanna Zimatore

**Affiliations:** 1 Clinic of Audiology & ENT, University of Ferrara, 44121 Ferrara, Italy; sdh1@unife.it; 2 Department of Life Science, Health, and Health Professions, Link Campus University, 00165 Rome, Italy; l.cardinali@unilink.it; 3 Heart Failure and Cardiac Rehabilitation Department, Faculty of Medicine and Dentistry, Medical University of Warsaw, 02-005 Warsaw, Poland; piotrhskarzynski@gmail.com; 4 Institute of Sensory Organs, 05-830 Nadarzyn, Poland; 5 World Hearing Center, Department of Teleaudiology and Screening, Institute of Physiology and Pathology of Hearing, 02-042 Warsaw, Poland; 6 Department of Special Education, University of Ibadan, Ibadan 200005, Nigeria; habins.city@gmail.com; 7 Department of Theoretical and Applied Sciences Applied Physics, Campus University, 22060 Novedrate, Italy

**Keywords:** hearing aids, cochlear implants, early hearing intervention, Africa

## Abstract

Background: The objectives of this scoping review were (a) to identify the most recent (in a 21-year span) literature information about hearing intervention programs in Africa and (b) to provide data on the intervention practices, policies and the factors prohibiting the larger diffusion of the hearing technologies in the African states. Methods: Queries were conducted via the PubMed and Scopus databases for the time window from 2005 to 2025. The mesh terms used were “hearing aids”, “cochlear implants”, and “hearing intervention Africa”. Only research articles and review papers were considered as good candidates. The standard English language filter was not used, so as to also identify information from non-English-speaking scientific communities and groups. Results: Data from eight papers were considered, reflecting the hearing intervention practices of six African states. These reports, although integral in themselves, examine different aspects of children’s hearing identification and grouping the information was not entirely feasible. It is assumed that since there are no organized or centralized NHS programs in the African states, the primary driver of hearing identification appears to be parental vigilance. The cochlear implant intervention is not very diffused mainly due to complex economic factors of the weak African economies. Anecdotal data refer to cultural bias versus hearing intervention technology, but this information needs further elucidation. Conclusions: The information on the African programs on hearing intervention policies is quite scarce, as in the case of African NHS. Within this context, it is very important to convince audiologists and ENTs from the African localized programs to publish their data in mainstream channels so that new information can be assessed.

## 1. Introduction

Childhood hearing loss constitutes a considerable public health issue, particularly in countries with low or middle incomes where access to early diagnostic and intervention services is restricted [1]. Early hearing detection and intervention (EHDI) programs, which include universal neonatal hearing screening (UNHS), timely hearing aid (HA) fitting, and cochlear implants (CIs), are essential to mitigate the impact of congenital hearing loss on speech, cognitive, and social development. In high-income countries, the systematic implementation of UNHS protocols has enabled the early identification of hearing impairment, facilitating prompt auditory intervention during critical periods of neuroplasticity. Early auditory access through hearing devices, coupled with consistent use, has been shown to significantly improve linguistic and developmental outcomes in affected children [2]. The earlier-implanted children are more proficient than later-implanted children in their phonological development [3,4]. Despite the established benefits, the implementation of UNHS and subsequent rehabilitative interventions across the African continent remains inconsistent and fragmented, with substantial disparities in program coverage, accessibility, and follow-up services.

A recent review [5] highlighted the severe lack of comprehensive NHS programs across the African continent, with available data reflecting localized initiatives in only a handful of countries and often limited to urban centers. Despite efforts to screen neonates using otoacoustic emissions (OAEs) and automated auditory brainstem response (AABR) technologies, the review underscored significant gaps in follow-up care, intervention strategies, and published outcomes, particularly regarding the use of devices such as HAs and CIs for children identified with hearing loss. Moreover, high loss-to-follow-up rates and infrastructural challenges, such as the shortage of trained audiologists and the social stigma associated with deafness, further compromise the effectiveness of these programs.

Given the critical role of early auditory intervention in improving developmental routes for children with hearing loss, it is essential to assess the extent to which HAs and CIs have been utilized among the African pediatric population over the past two decades. This scoping review complements the NHS and EHDI African data we have previously presented and focuses on the next step after the NHS, that is, on the hearing intervention practices available to the African children. The purpose of this work is to provide possible guidance for expanding access to effective auditory rehabilitation across the continent by identifying achievements, barriers, and gaps.

## 2. Materials and Methods

In various contexts, Africa is divided into the northern region and the sub-Saharan region, which includes 54 states. In this review, we considered the whole continent without any specific geographical divisions. As in the previous review analysis of the African neonatal hearing screening data, we have focused our investigation on the 84.45% of the total African population (1,532,073,577) that was measured in 2024 and reported in terms of the 25 most population-dense African states.

Since the present manuscript is the continuation of the paper on the African NHS and EDHI practices [5], we initially chose a 20-year window (2004–2024) to search for information. Due to the small manuscript yield of the initial searches, the year 2025 was added to the search window, which was conducted in April 2025 and followed the PRISMA 2020 guidelines. (See PRISMA Checklist Supplementary Materials Table S1). The queries utilized the following three keywords and phrases (mesh terms): “hearing aids”, “cochlear implants”, and “hearing intervention Africa”. The PubMed and Scopus databases were assessed, and research articles and review papers were considered as good candidates. One case report was also included due to the scarcity of information on the type of CIs used in the intervention practices. The review papers included in the study were assessed and controlled for possible overlap of information with the accepted research papers. The standard English language filter was not used, so as to identify possible information from non-English-speaking (i.e., French) scientific communities and groups. Papers related to hearing interventions outside the African continent were not considered. The inclusion and exclusion criteria are reported in Table 1.

Two independent reviewers reviewed the available material (38 manuscripts) and distilled the final number of eligible papers to 8. The quality criteria for the manuscript selection included: (i) the publication being in a peer-reviewed journal and (ii) the candidate paper clearly showing a scientific methodology style.

It should be noted that we were able to additionally identify (through the reference sections of the accepted manuscripts) a number of additional articles. These were published in African journals such as the *Pan African Medical Journal* and the *South Sudan Medical Journal*, which are not indexed in PubMed or Scopus. These papers were excluded from the submission evaluation filtering procedure. Theoretically, these sources (lacking an impact factor) would undermine the quality standards we have established for this scoping review, but considering the extreme lack of information, the presented data are reported in Appendix A and should be treated as anecdotal additional.

**Figure 1:**
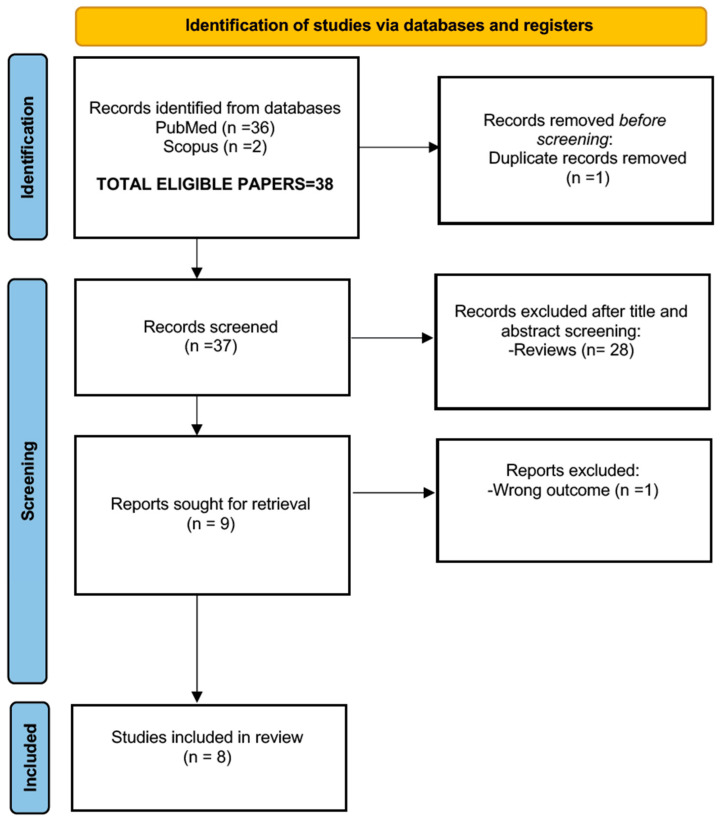
Flow diagram of the literature search, according to the PRISMA criteria (http://www.prisma-statement.org/, accessed 30 July 2024), with the steps followed in the manuscript selection procedure. After the application of the selection criteria, the initial 38 manuscripts were reduced to 8.

In Figure 1, the PRISMA flowchart process is reported. The candidate papers for this review are reported in Table 2.

## 3. Results

The data were classified alphabetically according to the country of origin. Considering that there are no organized hearing screening practices or early detection and hearing intervention programs in Africa [5], it is reasonable to postulate that the hearing deficits of the assisted children are identified by members of their families. This point, however, requires further elucidation and additional research.

Three of the eight papers selected were published in the last five years. Two papers were retrospective studies, and two were qualitative studies. The papers included data from one patient (a case report) to a maximum of 223 subjects. All these studies were conducted on a limited number of implanted subjects, except for one involving both CIs and HAs. Of the eight papers (for a total of 404 subjects), four reported data from severe SHL cases.

Table 3 synthesizes key themes across studies, noting both empirical results and modeled data.

**Table 3:** The 8 eligible papers after the filtering process: a comparative summary of key data points.

*n*	Study	Country	Age at Implantation	Post-Implantation Outcomes	Hearing Device Usage
1	El-Dessouky et al. (2019) [6]	Egypt	Mean: ~3 years	Improved auditory skills on the Egyptian Arabic scale	Consistent CI use with structured benefit
2	Mulwafu et al. (2025) [7]	Malawi	Median: ~4.8 years	Positive audiometric and speech perception results	CI was used consistently in a clinical setting
3	Adedeji et al. (2015) [1]	Nigeria	Not specified precisely; often delayed	Limited data; outcomes impacted by late diagnosis	Limited access and affordability issues
4	Jessop et al. (2007) [8]	South Africa	Range: 1–10 years	Parents report developmental progress	Regular CI use; variable based on support
5	Kanji et al. (2024) [9]	South Africa	Range: 2–6 years; delays are common	Emotional and social challenges highlighted	Use influenced by therapy access
6	Atiya et al. (2018) [10]	South Africa	12 months	Success with a hybrid device in partial hearing preservation	Consistent use of CI
7	de Beer et al. (2024) [11]	South Africa	Varies; includes early and late	High family impact; importance of support	Regular use tied to family support
8	Emmett et al. (2015) [12]	Sub-Saharan Africa *	Modeled early vs. late	CI is cost-effective in early implantation scenarios	Modeled consistent CI use

* Kenya, Nigeria, Malawi, South Africa, Rwanda, Uganda.

Analytically, the reported intervention policies are listed below, by African state, in alphabetical order.

### 3.1. Egypt

El-Dessouky et al. (2019) [6] conducted a validation study of the Arabic version of the auditory skills checklist using a cohort of 90 Egyptian children (mean age 36–72 months) with bilateral severe-to-profound prelingual hearing loss (since birth), all fitted with CIs. The children had no experience with CIs, but they were previously fitted with hearing aids. The paper offers no information on the time of HA fitting. The study reinforced the utility of culturally and linguistically adapted assessment tools in tracking auditory development. The cohort benefited from structured intervention and follow-up protocols, demonstrating significant post-implant auditory skills development within a 3-year period. Detailed scores of linguistic development were reported in terms of detection, identification, short-term auditory memory, supra-segmental discrimination, segmental discrimination, and linguistic auditory processing.

### 3.2. Malawi

Data from the state of Malawi are available from Mulwafu et al. (2025) [7], detailing the early outcomes from the country’s first cochlear implantation program. This paper presents the most detailed information about the linguistic evaluation of the implanted children. The paper reports data from 17 patients who received a CI from 2014 to 2022. The subjects presented postlingual HL and received the Synchrony FLEX 28 implant from Medel. Deafness was identified at 8.7 ± 6.3 y and the implantation was conducted at 10.8 ± 4.7 y. Audiometric and speech perception improvements were observed, demonstrating promising clinical results despite infrastructural limitations. The most significant results were obtained in the Monosyllabic Tronchee Polysyllabic test (MTP), where the authors declared: “14 children (82.3%) achieved scores of 20 or more out of the maximum 24. The remaining 3 children achieved scores of 12, 2, and 1. In the MTP syllables test, 12 children (70.5%) achieved scores of 20 or more out of 24. The remaining 5 children achieved scores of 18, 18, 17, 7, and 1.”

### 3.3. Nigeria

In Nigeria, a retrospective descriptive study conducted by Adedeji et al. (2015) [1] reviewed 223 children with congenital and early-onset childhood hearing loss. The study highlighted significant delays (not defined in the paper) in diagnosis and intervention, largely due to limited audiological services and public awareness. The primary interventions reported included HAs and CIs, though access remained restricted to urban tertiary facilities. This underscores the need for community-based screening and early intervention programs.

Due to the scarcity of the reported material, we are presenting in Appendix A additional from one of the authors (A.A.). The data were derived from a Government Northern Central Hospital in Nigeria, and show a constant flat rate of HA requests from 2021. Data on CIs are also presented. It should be noted that the northern part of Nigeria consists of more Hausas who hold the belief that whatever happens to people is from God, which may reduce their motivation to actively pursue hearing interventions such as acquiring HA, as well as other health-related treatments. These anecdotal conclusions still need validation through questionnaires or other means.

### 3.4. South Africa

South Africa had the most extensive coverage across the literature, regarding data on HAs and CIs, spanning 17 years and represented by four articles. According to Emmett et al. [12], South Africa is currently the only country within the low/middle-income context of sub-Saharan Africa that has established a national CI program.

Jessop et al. [8] described 45 cases from the Pretoria CI program. Their paper was based on questionnaire data from queries sent to 71 families, of which only 45 were returned. The age of implantation was varied from 12 months to 10 years. The main aim of the paper was to describe the CI outcomes, as perceived by their parents. The reported causes of hearing loss, in declining order, were unknown, meningitis, genetic syndrome, prenatal complications, birth trauma, rubella, and cytomegalovirus. In terms of linguistic development, the authors declared, “More children in this study were using single words than more complex word combinations, however, an even larger group was using mature sentence forms. This indicated that in the present cross-sectional study, more children were in either the earlier, or the more advanced stages of language acquisition than in the middle stages”.

In Soweto, South Africa, a CI program was developed at the Chris Hani Baragwanath Academic Hospital. This is one of the few fully state-funded programs in the country and targets a poor population [10]. The paper provides data from a single CI case, using Cochlea’s Hybrid CI24REH device on a male subject 12 months old. In terms of linguistic development, the authors reported that, according to the results of the 34-month-old Rossetti Infant–Toddler Language Scale, language expression was measured at 15 months and understanding at 12 months. The subject scored between 30 and 33 months on both language expression and understanding when it was repeated at 44 months, indicating that his language delay had considerably decreased during the preceding 12 months.

De Beer et al. [11] explored family perspectives post-implantation, emphasizing the social and emotional dimensions of intervention in a sample of 29 parents of 26 pediatric CI recipients (younger than 18 years) who had been implanted for at least 12 months at the time of data collection. Concept maps were derived in order to weigh the impact of various factors on the life of the implanted subjects and according to the authors “The maps revealed six important areas of social impact, namely (i) Financial Outlay and Supports; (ii) Education and Therapy; (iii) Responsibilities and Sacrifices; (iv) Extended Family and Community; (v) Spouses and Siblings and (vi) Achievements and Enrichments.”

Lastly, Kanji et al. [9] conducted qualitative interviews with parents of children who received CIs, stressing the challenges related to costs and post-operative support.

Collectively, the South African studies document a structured, albeit resource-limited, cochlear implantation infrastructure, with growing attention to holistic, family-centered care.

### 3.5. Sub-Saharan Area (Kenya, Malawi, Rwanda, and Uganda)

These countries were primarily examined in a cost-effectiveness analysis by Emmett et al. [12], comparing the implementation of CIs and deaf education. While specific sample sizes per country were not disaggregated, the study supported the feasibility and value of cochlear implantation within these low-resource settings when paired with sustained rehabilitation and education. The interesting part of the study was that local reference clinicians (who were also co-authors) collected national data regarding CIs, but the analytical information is not available in the paper.

The authors reached interesting and optimistic conclusions: (i) South Africa has the most developed socioeconomic structure in the sub-Saharan group, which makes cochlear implantation extremely cost-effective; (ii) Nigeria shows that CIs can reach the cost-effective threshold and in order to reach 30% of the estimated children in need, the current Nigerian implant program, which only provides five implants annually, must increase by more than 500%; (3) the other countries in the study show the potential to extend their CI programs to rural areas that have not historically had access to this technology. To create strong national CI initiatives, philanthropic, academic, and commercial partnerships are required in Kenya, Rwanda, Uganda, and Malawi. In these growing economies, device cost and related maintenance have a significant impact.

## 4. Discussion

**Figure 2:**
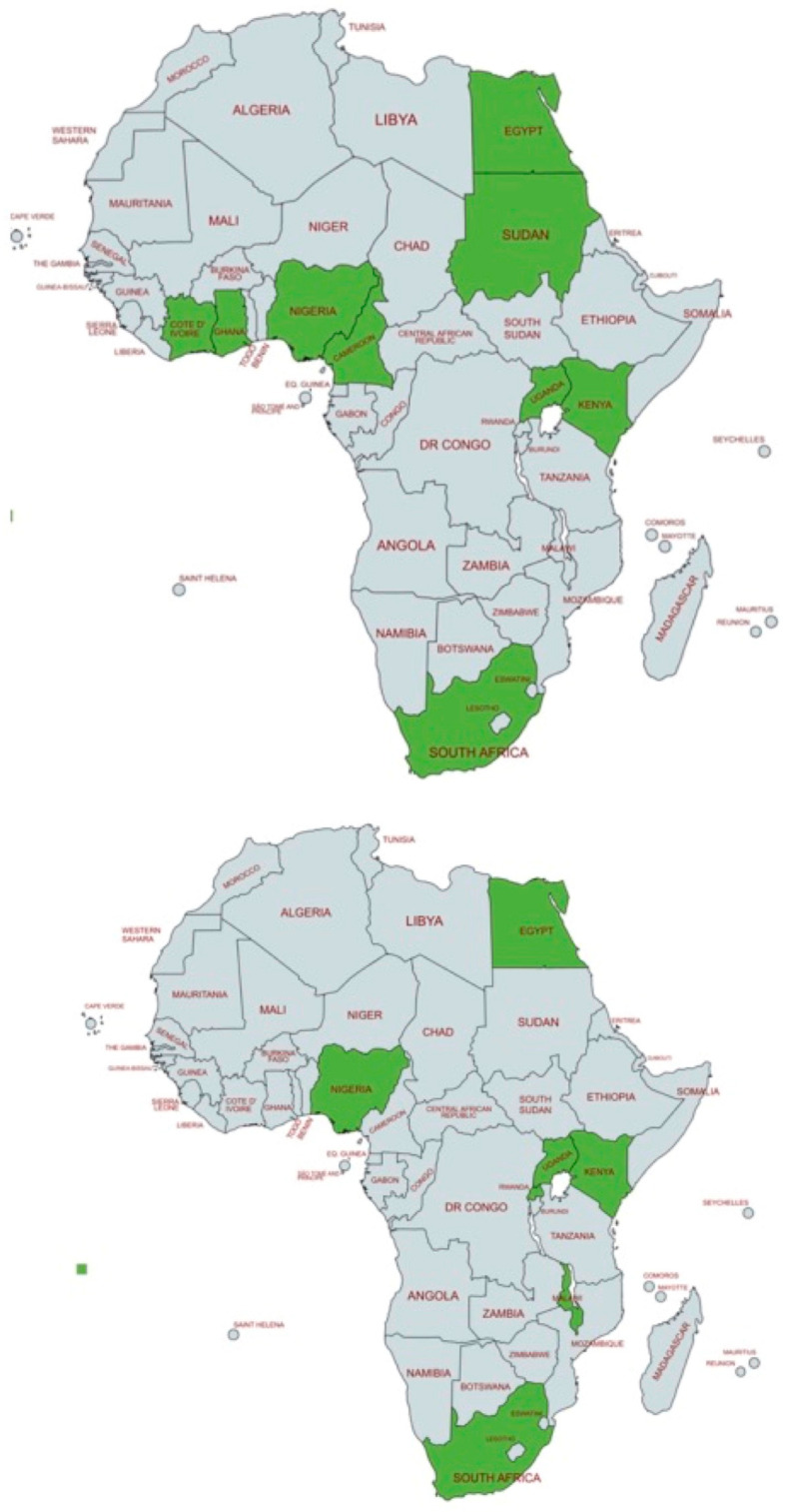
Comparison of the African states reporting newborn hearing screening (top) and those reporting hearing intervention policies (bottom). Countries with active hearing programs or hearing intervention activities are shown in green. The map at the top shows the countries where hearing screening data are reported in the literature [5]. The map at the bottom shows the countries where information on hearing intervention policies is reported, including Egypt, Kenya, Malawi, Nigeria, Rwanda, South Africa, and Uganda.

The aim of the paper was to collect as much information as possible on the reported hearing intervention policies in the African states. Due to the lack of organized neonatal hearing programs and other relevant information, the data of this review refer to intervention practices and not intervention policies and strategies. The data collected show a significant scarcity of information, in terms of the selected practices, the intervention timing, intervention complications, etc. In order to elucidate better this aspect, we present in Figure 2 geographical data regarding the status of African hearing screening and hearing interventions: (i) the top map presents the African states who have reported neonatal hearing screening (NHS) practices (data from a previous review paper by [5]), and (ii) the bottom map shows the African states offering hearing intervention programs: Egypt, Kenya, Malawi, Nigeria, Rwanda, South Africa, and Uganda. All active programs are shown in green color.

This figure is designed to illustrate the lack of overlap between early hearing detection efforts and actual hearing intervention practices. Notably, regions with reported hearing screening activities are often the same regions where interventions such as HAs and CIs are implemented, suggesting that without early identification of hearing loss, further intervention is unlikely to occur. Otherwise, the fact that the data from the two maps do not overlap well suggests that some partial intervention exists, even without any hearing screening basis. Based on the articles we have found in non-mainstream impact factor journals, we postulate that data on the clinical practices in the areas of hearing screening and intervention are not reported in these channels, and they are not easily traceable.

To better clarify this aspect, we show in Appendix A clinical data on HAs and CIs, which are not reported in the standard PubMed and Scopus databases. This pattern is not unexpected, as many clinical settings generate data that remain unpublished in high-impact journals—potentially due to the economic constraints of open access publishing—or are instead disseminated through non-indexed local publications. Similar underreporting has been documented in other regions globally, including within European countries, where published data on hearing screening remain limited, as highlighted in a recent study by our group [13].

The totality of collected information about the hearing intervention practices in the African states cannot be aggregated in meaningful groups, as it is quite sporadic. Unfortunately, the collected information does not allow (i) a comparison of results between the various African states, (ii) the identification of trends in government intervention policies, or (iii) the identification of the missing features in the intervention programs. Therefore, the reported outcomes are difficult to compare with the data from interventions in other parts of the world.

Nevertheless, several aspects of the hearing intervention policies can be minimally discussed, as follows.

### 4.1. Intervention Times

Since there are no organized hearing screening programs on the African continent, it has been postulated that children with hearing deficits eventually get assisted by members of their families, who probably monitor their hearing behavior. This is typical behavior in the Western world [14,15]. Since there is a lack of specific information on this issue, the postulate was based on anecdotal information from one of the co-authors (A.A.). The aspect of the intervention timing point is extremely important for any intervention strategy and therefore requires additional elucidation studies.

The majority of the available data with reference to CIs do not indicate the time when the subjects were implanted after being fitted with an HA. Data from papers presenting the actual age of the assisted patients with a CI are available from Egypt, Malawi, and South Africa. The minimum ages of the implanted subjects range from 10 months (South Africa) to 10.8 years (Malawi). The Egyptian data refer to a minimal age of 36 months, but for subjects who were already fitted with an HA. Although the Malawi estimates are derived from a starting pilot project, it must be noted that they are significantly larger than the recommended standards used in most European and American early detection and intervention programs that start an intervention at 6 months [14,15,16]. Anecdotal data in Appendix A suggest that in Nigeria and Tanzania, intervention policies are applied as early as 2 years.

### 4.2. Factors Inhibiting the Hearing Intervention Policies

In the previous review paper on African neonatal hearing screening, we identified a series of obstacles related to the distribution of the new screening technologies, primarily related to economic, cultural, and religious beliefs. The majority of the papers in this review (except the paper on the sub-Saharan countries by Emmett et al. [12]) do not mention any of those factors, but some anecdotal information is presented in Appendix A.

The costs of the CI part of the intervention policies are seen as the main factor inhibiting a wider distribution of assistance services from central to rural areas. This is a complex issue to affront in a third-world context, because, as Emmett et al. [12] show, the actual costs of a CI intervention include not only the cost of the device but also the costs related to hospitalization and post-intervention procedures.

Regarding the anecdotal resources:

(i) One of the authors of this review (A.A.), who has colleagues working in Nigerian hospitals, has provided anecdotal evidence suggesting that in northern Nigeria, religious beliefs significantly impede the diffusion of hearing intervention policies (see the Section 3.3). Since there are cultural issues related to the diffusion of the screening strategies, it can be safely assumed that the same concepts will impede the application of HAs and CIs to the general population. Nevertheless, these aspects need to be better clarified with well-defined questionnaires.
(ii) Anecdotal data from Senegal suggest that the costs of the screening procedures and the intervention devices are strongly prohibiting the diffusion of the intervention solutions to the general population. Again, these aspects need to be better clarified with well-defined questionnaires.

### 4.3. Technologies Used in the Hearing Intervention

From the eight papers included in the review, only one, referring to the Egyptian data, made a reference to HAs prior to implantation. Additional references were not reported, so the names of the devices and the relative clinical and hardware protocols are not known.

In terms of CI brands, the information was specified in only two papers: the Cochlear Hybrid CI24REH model in [10], and MEDEL SONATA TI and Advanced Bionics (HiRes 90K) in [6].

### 4.4. Possible Limitations of the Study

Since the number of papers in this review is low, the overall conclusions regarding the status of the African intervention policies might be biased and inconclusive.

Additionally, since we have found a number of publications not indexed in the PubMed or Scopus databases, it is reasonable to assume that there might be others as well, which we did not identify. These sources, although treated as anecdotal, might contain information useful in the context of this review.

## 5. Conclusions

Across these African nations, cochlear implantation emerged as the dominant intervention for severe-to-profound hearing loss. However, disparities persist in terms of access, infrastructure, and post-implantation support. Nigeria and Egypt report relatively higher sample sizes and infrastructure, while South Africa demonstrates multidimensional research on clinical and psychosocial outcomes. Kenya, Rwanda, Uganda, and Malawi represent emerging frontiers in cochlear health services, warranting further investment and research. A regional strategy to standardize care, expand audiology services, and integrate family-centered rehabilitation models is essential for sustainable impact. To create strong national HA and CI initiatives, philanthropic, academic, and commercial partnerships are strongly required.

## Figures and Tables

**Table 1:** Selection criteria.

**Inclusion Criteria**
Article type: research, review papers, and eligible case reports Article scope: article reports on hearing intervention practices—including hearing aids and cochlear implants—among African children Participants: children (<18 y) Area of application: articles that conducted research in the field of deaf mitigation by HAs and CIs Language: English, French Publication period: last 21 years (considering only 3 months of 2025)
**Exclusion Criteria**
Article type: opinions, commentaries, letters to the editor, or conference papers Article scope: not related to HAs and CIs Participants: animals, adults Area of application: not related to hearing intervention practices (classified as “wrong outcome”), hearing interventions outside the African territory Language: none Publication period: published >21 years ago

**Table 2:** The 8 eligible papers after the filtering process: The national data are presented in alphabetical order.

*n*	Country	Type of Hearing Impairment	Sample Size (*n*)	Period of Data Collection	Type of Paper	Author (First)	Publication Year
1	Egypt	Bilateral prelingual severe to profound SHL	90	July 2015–January 2017	Validation study (cross-sectional cohort)	El-Dessouky [6]	2019
2	Malawi	Severe to profound SHL	19	2014–2022	Case series study	Mulwafu [7]	2025
3	Nigeria	Congenital and early-onset childhood HL	223	January 2008–December 2013	Retrospective descriptive study	Adedeji [1]	2015
4	South Africa	Congenital and progressive HL	45	2004 (retrospective)	Cross-sectional quantitative survey	Jessop [8]	2007
5	South Africa	Severe to profound SHL	7	2022–2023	Qualitative exploratory study (mothers interviewed)	Kanji [9]	2024
6	South Africa	Bilateral profound SHL with cochlear malformation	1	2015	Case report	Atiya [10]	2018
7	South Africa	Not explicitly specified, focus on the family experience post-implant	19 CI	COVID-19 epidemic period, (2020–2022, not specified precisely)	Qualitative conceptual framework (by parents)	de Beer [11]	2024
8	Sub-Saharan Africa *	Severe-to-profound congenital SHL	Not specified	2012	Economic evaluation study	Emmett [12]	2015

* Kenya, Nigeria, Malawi, South Africa, Rwanda, and Uganda.

## Data Availability

The original contributions presented in this study are included in the article/Supplementary Material. Further inquiries can be directed to the corresponding author.

## Supplementary Materials

The following supporting information can be downloaded at: https://www.mdpi.com/article/10.3390/children12070864/s1, Table S1: PRISMA 2020 Checklist. Ref. [17] is cited in Supplementary Materials.

## Appendix A

### Appendix A.1. Anecdotal Supplemental Information from Nigeria

One of the co-authors, A.A., reports the following unpublished HA (* as of May 2025) and CI trend data from Nigeria in a Government Central Northern Hospital. Usually, the fitting procedures are conducted in external clinics, and then the patients must come back to the hospital to undertake follow-up controls. As the data show, very few cases come back, and in this context, the information about problems related to HAs is not well defined. Since there is no state assistance for buying an HA device, the number of purchases is low (in comparison to referrals), but the data within the last 3 years show a moderate increase in purchases. Even in these cases, the follow-up rates are significantly low.

**Table A1:** The second column indicates the number of referrals for a hearing aid (HA), the second the number of purchased devices, and the third column the number of patients who have come back for a follow-up.

Year.	HA Referral.	Purchase.	Follow-Up
2021.	3.	-	-
2022.	19.	6.	1
2023.	13.	2.	1
2024.	19.	3.	1
2025. *	11.	11.	1

In terms of CIs performed in an external clinic in a time window of 12 years (2012–2024), 137 patients have received a single implant. The demographic characteristics of the patients were 2–3 y (26 cases), 4–6 y (37 cases), 7–10 y (14 cases), 10–13 y (8 cases), and >13 ys (52 cases). Additional information was not available.

### Appendix A.2. Anecdotal Supplemental Information from Senegal

The data were presented in a paper by Lame et al. [18] in the *Pan African Medical Journal*. The paper examined the possibilities and the impediments of cochlear implantation in Senegal, the main factor being the high cost of the medical procedures offered in a non-insurance context.

The limitations regarding the application of hearing intervention policies are summarized by the authors. Senegal has a variety of diagnostic methods, such as auditory brainstem response and otoacoustic emissions; however, they are primarily located in the capital, Dakar, and are very expensive, particularly for those without health insurance. The anticipated cost of a full assessment for deafness is XOF 500,000 (EUR 765). This is equivalent to five and a half times the average monthly gross salary in Senegal. The Ministry of Economy, Planning, and Cooperation of Senegal reported that the average gross monthly pay in 2019 was XOF 89,730 (EUR 138). In most cases, employers or insurance companies can cover complementary examinations and surgery for employees and their families.

However, the patient still bears the burden of purchasing the implant, which is the costliest component. It is necessary to provide CI candidates and their families with financial support. There is a technological platform accessible for cochlear implantation. However, currently, only three places have implemented this type of intervention. There are not many speech therapists in the nation. There are currently fewer than ten of them, and they are responsible for all patients’ rehabilitation across all specializations.

### Appendix A.3. Anecdotal Supplemental Information from Tanzania

The data were presented in a paper by Kahinga et al. [19], in the *South Sudan Medical Journal*, from 39 patients (2–55 y with a mean age of 4.7 y), who were implanted in the Muhimbili National Hospital. The demographic characteristics of the patients were 2–3 y (24 cases), 4–5 y (13 cases), and >5 (2 cases). Interestingly, the authors refer to a case of a 55 y patient without any reason for why this case was included in the pool with the other data. The main causes of hearing loss were ototoxicity and birth asphyxia.

Thirty-seven patients presented with prelingual bilateral profound hearing loss. Due to the high costs of the CI, 37 out of the 39 patients were implanted with a single device. The support for the costs of the implants came from the Tanzanian government.

Data for post-operatory speech performance were available for only one case, who moved from 0 to 36 points on the Infant–Toddler Meaningful Auditory Integration Scale (IT-MAIS) over a period of 18 months, post-implantation.

This paper is the only one of the papers in this review that refers to CI post-operative complications. The authors declare that the described symptoms were “facial weakness, which was the commonest, followed by skin infection at the surgical site which led to extrusion of the device. Other complications such as meningitis, was not reported and this could be attributed to the provision of pneumococcal polysaccharide vaccine and Haemophilus influenzae vaccines prior surgery”.
